# 4269 Frequent emergency department use among homeless individuals seen in emergent care: High risks of opioid-related diagnoses and adverse health services utilization outcomes

**DOI:** 10.1017/cts.2020.394

**Published:** 2020-07-29

**Authors:** Ayae Yamamoto, Lillian Gelberg, Yusuke Tsugawa, Gerald Kominski, Jack Needleman

**Affiliations:** 1 David Geffen School of Medicine at UCLA; 2 UCLA Fielding School of Public Health

## Abstract

OBJECTIVES/GOALS: Using multi-state discharge data, to identify predictors of frequent emergency department (ED) use among the homeless patients seen in emergent care, and to compare frequent versus less frequent homeless ED users for their risk of serious health services utilization outcomes. METHODS/STUDY POPULATION: Based on the State Emergency Department Database and the State Inpatient Database, homeless individuals (n = 88,541) who made at least one ED visit in four states (Florida, Maryland, Massachusetts, and New York) in 2014. In this retrospective cross-sectional analysis, patient-level demographic and clinical factors were assessed as predictors for increased ED use. Risks of opioid overdose, opioid-related hospital admission/ED visit, in-hospital mortality, mechanical ventilation, and number of hospitalizations were compared between individuals with 4 or more vs. 2-3 vs. 1 ED visit(s), adjusting for potential confounders including hospital fixed effects (allowing for within hospital comparisons). RESULTS/ANTICIPATED RESULTS: Higher rates of ED use were associated with Medicare coverage <65; primary diagnosis of alcohol abuse, asthma, or abdominal pain; and co-morbidity of alcohol abuse, psychoses, or chronic pulmonary disease. Individuals with ≥4 visits had significantly higher adjusted risk of opioid overdose (3.7% vs. 1.2% vs. 1.0%), opioid-related hospitalizations/ED visits (17.9% vs. 8.5% vs. 6.6%), mechanical ventilation (9.8% vs. 7.0% vs. 4.7%), and greater # of hospitalizations (3.2 vs. 1.3 vs. 0.8) compared to individuals with 2-3 or 1 ED visit. Individuals with ≥4 and 2-3 ED visits had similar but increased risks of in-hospital mortality compared to individuals with 1 ED visit (2.8% vs. 2.8% vs. 2.3%). DISCUSSION/SIGNIFICANCE OF IMPACT: Homeless patients who were high ED users were more likely to be hospitalized and have other adverse outcomes. These findings encourage targeted interventions (i.e. housing) for the high-utilizer homeless population to reduce the burden of serious outcomes and costs for the patient and society.

